# Daily Dispo Documentation Improves Patient Flow and Discharge Efficiency

**DOI:** 10.7759/cureus.88814

**Published:** 2025-07-26

**Authors:** George Bechir

**Affiliations:** 1 Hospital Medicine, Franciscan Health Munster, Munster, USA

**Keywords:** daily progress note, discharge planning, dispo documentation, expected discharge date, hospitalist documentation, inpatient care coordination, length of stay, patient flow, quality improvement, readmission prevention

## Abstract

“Dispo,” short for disposition, refers to the anticipated plan for a hospitalized patient’s discharge, including timing and destination. Reducing hospital length of stay requires more than discharge orders and case management rounds. It demands daily, structured planning embedded in the clinical workflow. This review explores the underused yet high-impact strategy of “daily dispo documentation,” in which hospitalists explicitly document five key discharge planning elements in each progress note. This framework has been adopted by some institutions to improve discharge efficiency. These elements are expected discharge date, consultant communication, patient and family communication, reason for not discharging today, and planned discharge destination. Each element plays a distinct and actionable role. Setting and updating the expected discharge date creates a shared mental target that drives task completion and team alignment. Capturing consultant communication ensures clarity and resolution of pending recommendations, a common source of delays. Documenting patient and family discussions strengthens readiness and engagement, reducing confusion and post-discharge errors. Recording the reason for not discharging today transforms the note into a daily operational audit by making barriers visible and prompting immediate problem-solving. Finally, stating the planned discharge destination early allows social work and case management to arrange placements proactively, reducing failures on the day of discharge. Framed within established models such as IDEAL (initiating, diagnosing, establishing, acting, and learning), which involves patients and families in discharge planning, SAFER, a set of steps to improve patient flow, and Red2Green, which minimizes unnecessary hospital days, this approach shifts documentation from passive recordkeeping to active discharge coordination. Hospitals that implement daily dispo notes have demonstrated reductions in length of stay, smoother care transitions, improved patient satisfaction, and operational and financial benefits. As healthcare systems aim to improve efficiency while maintaining safety and patient-centered care, embedding dispo elements into the daily note offers a scalable and evidence-based solution rooted in frontline workflow.

## Introduction and background

Effective and safe patient transitions are critical for modern healthcare. Prolonged hospital stays and unplanned readmissions not only increase healthcare costs but also expose patients to avoidable risks such as infections, deconditioning, and care fragmentation, making discharge planning a key quality and safety priority. A powerful and often overlooked tool in this process is daily disposition documentation (“dispo”), referring to the daily explicit documentation of a patient’s anticipated discharge plan within the hospitalist’s (inpatient physician’s) routine medical record. This proactive dispo is distinct from a final discharge summary, serving instead as a forward-looking plan that prompts specific actions like securing post-discharge services or resolving specialist consults. Its consistent use directly addresses significant healthcare burdens.

For instance, it helps manage length of stay (LOS). Prolonged LOS escalates healthcare costs and increases patient risks like hospital-acquired infections and functional decline [1]. Dispo documentation aids in proactively identifying and resolving logistical bottlenecks that delay discharge, thereby optimizing bed use and patient flow [1,2]. Similarly, it helps reduce high readmission rates, a major indicator of fragmented care and inadequate post-discharge support. By embedding continuous communication and preparation within the daily note, dispo documentation improves care coordination, reducing preventable readmissions [1,3]. Furthermore, it enhances patient experiences by reducing anxiety, improving understanding of their care plan, and fostering trust in the team [4,5].

This approach aligns with established improvement frameworks: IDEAL (Include, Discuss, Educate, Assess, Listen), which emphasizes engaging patients and families in discharge planning; SAFER, a bundle of steps to improve patient flow; and Red2Green, which minimizes unnecessary hospital days by tracking progress and delays. By embedding comprehensive discharge planning into daily workflow, dispo documentation promotes efficient, safe, and patient-centered hospital care. This narrative review synthesizes existing literature and established conceptual frameworks to explore the multifaceted impact of incorporating disposition elements into hospitalist progress notes.

## Review

Methodology

A targeted literature search was conducted across PubMed, PMC, and Google Scholar databases, as well as through professional organization websites, such as the Agency for Healthcare Research and Quality (AHRQ) and the Society of Hospital Medicine (SHM). Additional grey literature and policy reports were identified through the National Health Service (NHS) and institutional quality improvement publications. The search covered articles published from January 2000 through May 2025, in order to capture both foundational frameworks and contemporary evidence.

Search terms included the following combinations: “discharge planning progress note”, “hospitalist documentation”, “expected discharge date length of stay”, “readmission reduction strategies”, “patient flow management”, “electronic health record nudges”, “audit and feedback in healthcare”, “hospitalist compensation models”, “inpatient discharge documentation”, “daily patient disposition”, “care transitions in progress notes”, “patient-centered discharge”, and “health equity in hospital discharge”. Boolean operators and MeSH terms were applied where appropriate to broaden or refine results. Filters for English language and adult inpatient settings were used where available.

Inclusion criteria were as follows: peer-reviewed journal articles, systematic reviews, randomized controlled trials, cohort studies, and high-quality organizational reports that addressed at least one of the following domains in adult inpatient hospital care - the impact of discharge documentation on length of stay, readmissions, patient satisfaction, workflow efficiency, operational outcomes, legal and regulatory compliance, or health equity. Both United States and international studies were eligible. Only articles written in English were included.

Exclusion criteria were studies focusing exclusively on pediatric, psychiatric, or rehabilitation settings, single-case reports, editorials, and commentaries without original data or substantive review.

The initial search yielded approximately 220 articles and reports. After screening titles and abstracts for relevance, 86 full-text articles were reviewed in detail. Ultimately, 39 sources were included in this narrative review, comprising 29 peer-reviewed studies, six systematic reviews, and four organizational guidance documents. Priority was given to studies indexed in PubMed with DOIs and clearly described methods. Given that many included studies were observational or single-center, we acknowledge the potential risk of bias and limited generalizability in the underlying evidence base. As a narrative review, our intent was to synthesize key themes and frameworks rather than conduct a formal systematic assessment of individual study quality.

This approach ensured a comprehensive yet focused synthesis of the evidence supporting the clinical, operational, and patient-centered value of structured disposition documentation.

Conceptual frameworks guiding "dispo in the progress note"

Integrating detailed dispo elements aligns with several established frameworks aimed at improving patient flow, safety, and communication. The IDEAL (Include, Discuss, Educate, Assess, Listen) Discharge Planning model from the AHRQ emphasizes partnering with patients, covering essential areas, using plain language, checking understanding, and responding to concerns [5]. Dispo elements like "patient and family communication" and "planned discharge destination" support these goals by fostering daily engagement and transparency. The SAFER (Senior review, All patients have an expected discharge date, Flow of patients, Early discharge, Review of patients with extended stay) Patient Flow Bundle and Red2Green Days operational frameworks focus on minimizing unnecessary delays. They encourage identifying each patient day as either a "Red" (delayed) or "Green" (productive) day [6]. Documenting the "Expected Date of Discharge (EDD)" and "Reason for Not Discharging Today" directly supports this classification, helping care teams address delays in real time. EHR integration strategies, like the Connect Care EDD Workflow, reflect a modern shift toward embedding discharge planning in the electronic health record (EHR). Dispo elements are suited for EHR templates, promoting early EDD entry, prompting necessary actions, and enhancing visibility of the discharge plan for the entire team.

Element-by-element evidence: the core impact of "dispo in the progress note" components

Consistently recording and updating an EDD builds a shared mental model across care teams. It encourages goal-oriented planning by prompting daily reviews of necessary steps, like tests and post-acute coordination. Evidence from the SAFER Patient Flow Bundle shows that clearly communicated EDDs correlate with reduced LOS and better bed turnover [6]. It also highlights when medical readiness is achieved, but delays persist due to system issues [4].

It is important to note that while dispo documentation contributes significantly to improving discharge processes, its specific impact can be challenging to isolate given its frequent integration into broader, multi-component quality improvement initiatives. Documenting consultant communication addresses a common discharge barrier, which is delays in specialist input. This documentation confirms whether the appropriate consultants were contacted, whether their plans align with the expected discharge date, and whether their recommendations have been received. Such clarity promotes accountability and minimizes delays caused by unresolved consultant plans. Similarly, documenting updates shared with patients and families improves discharge readiness by ensuring they are informed about the expected discharge date, barriers to discharge, and planned destination. This transparency helps patients and families remain engaged and better prepared for discharge. A randomized controlled trial demonstrated that structured discharge education using teach back methods significantly reduced hospital readmissions and improved adherence to post-discharge self-care among patients with heart failure [7]. This entry encourages real-time identification of delays, reflecting the Red2Green concept. Whether due to pending tests, placement issues, or consultant decisions, documenting these delays helps teams spot and resolve recurring bottlenecks [8]. Stating the planned destination early (e.g., home, SNF, rehab) allows case managers to arrange services in advance. This avoids last-minute placement issues and reduces denials or delays from post-acute providers [9,10].

Financial and incentive levers

Dispo documentation can be promoted through incentives. Some hospitals link bonus payments to note quality and completeness (e.g., EDD, patient discussions), aligning provider effort with system goals [11]. Participation in CMS programs like MIPS benefits as well. Comprehensive dispo documentation improves metrics tied to care coordination, experience, and readmissions, all of which affect MIPS scoring and reimbursement [12].

Implementation lessons

Leaders must prioritize change management. Leadership support, standardized rounds, and EHR templates can help integrate dispo into workflows [13]. Audit-feedback loops that review documentation and provide direct feedback to providers reinforce expectations and drive improvement [14]. EHR nudges - such as default fields, reminders, or mandatory EDD prompts - make dispo documentation easier and more reliable [15]. Practical challenges also need to be considered, such as some clinicians being reluctant to change their usual habits or feeling that the new documentation takes more time. Developing and maintaining EHR templates can also require extra time and technical support from IT teams. These challenges can often be addressed by explaining the benefits clearly, involving frontline staff in planning, and starting with small, easy-to-implement steps to build acceptance.

Updated gaps and future research

Randomized controlled trials showing that dispo documentation alone reduces readmissions are lacking [16]. There is also limited economic data on the cost-effectiveness of EDD dashboards and related tools, which hospitals need to justify investments [17]. Equity outcomes remain unclear, as recent research has highlighted disparities in the quality and language concordance of discharge instructions for patients with limited English proficiency [18]. More work is needed to ensure that dispo benefits all patients equally, regardless of language, literacy, or socioeconomic status.

Comparison to other discharge tools

Common tools like bedside whiteboards, huddles, or interdisciplinary rounds often lack consistency and traceability. Whiteboards may be outdated or invisible to certain staff. Verbal handoffs rely on memory and presence. Dispo documentation, in contrast, offers a shared, written plan updated by the decision-maker-the hospitalist. It is always available for nursing, case management, or covering providers to reference, reducing confusion and improving continuity [13, 19].

Multidisciplinary coordination benefits

Clear dispo documentation enhances coordination. Case managers can initiate SNF referrals earlier [10]. Therapists can assess patients sooner for home readiness [6]. Nurses can teach patients and families using the documented plan [5]. Studies confirm that structured documentation fosters shared understanding and shortens hospital stays [2]. These team-based approaches have evolved over time in response to clinical, operational, and policy pressures, as described in the following section on their historical and global development.

Evolution and global perspectives

The practice of documenting discharge planning in a structured, forward-looking manner has evolved considerably over the past few decades, shaped by both clinical and policy imperatives. In the mid-to-late 20th century, hospital discharge planning was often informal, relying on verbal updates and ad hoc coordination between physicians, nurses, and social workers. However, with increasing evidence linking prolonged hospital stays to adverse outcomes such as infections, functional decline, and readmissions, formalized discharge processes began gaining prominence. The introduction of Medicare’s Prospective Payment System (PPS) in 1983, which incentivized hospitals to reduce LOS, further accelerated attention to early discharge planning and documentation practices [20]. More recently, the Hospital Readmissions Reduction Program (HRRP), implemented by the Centers for Medicare & Medicaid Services (CMS) in 2012, tied hospital reimbursement to 30-day readmission rates for certain conditions, highlighting the critical role of thorough, timely, and transparent discharge planning [21]. These regulatory and financial pressures have spurred hospitals to embed disposition planning into daily workflows, making structured progress note documentation a key strategy in optimizing patient flow and ensuring safe transitions of care. Similarly, international experiences have validated these approaches. In the United Kingdom, a study evaluated the SAFER patient flow bundle and Red2Green methodology in a neurosurgery department, reporting improved patient throughput and reduced delays in discharge [22]. Likewise, a Brazilian tertiary-care hospital validated these tools, reducing median hospitalization from 19 to 14.2 days after implementation [23]. These findings illustrate that embedding disposition planning into routine documentation is a globally effective, evidence-based strategy that enhances team coordination and patient-centered care.

Impact on clinician workload and burnout

While structured discharge documentation enhances patient flow and care coordination, its effect on clinician workload and burnout is an important consideration. Excessive documentation requirements are a well-documented contributor to physician stress; however, emerging evidence suggests that structured, team-oriented discharge planning tools can actually improve efficiency and reduce duplication of work [24]. By making the discharge plan explicit and visible to the entire care team, hospitalists report spending less time fielding redundant questions and repeating information to different stakeholders, which improves overall workflow efficiency [25]. Moreover, studies have shown that streamlining and standardizing progress notes through templates and checklists improves physician satisfaction, reduces cognitive burden, and allows clinicians to focus more on direct patient care [26]. In addition, strategies such as automating certain fields or incorporating AI tools, as discussed in the Technological Innovations section, can further reduce the documentation burden while maintaining efficiency and supporting clinician well-being. These findings indicate that rather than adding to documentation fatigue, structured disposition documentation, when thoughtfully implemented, can support clinician well-being by improving workflow transparency and reducing inefficiencies.

Technological innovations and EHR integration

The adoption of structured discharge documentation has been further supported by innovations in EHR systems and related technologies. EHR-based tools, such as mandatory fields for expected discharge date, automated reminders, and dashboard displays, have been shown to improve the completeness and timeliness of discharge planning documentation [27]. These technological interventions enhance team situational awareness by making the discharge plan visible and accessible in real time to all care team members. Additionally, incorporating audit-and-feedback mechanisms within EHR workflows has been associated with measurable improvements in adherence to best practices and care quality [28]. More recently, research has explored how artificial intelligence (AI)-enabled predictive models embedded in the EHR can assist clinicians by estimating discharge readiness and highlighting unresolved barriers, offering a promising avenue for augmenting discharge efficiency while minimizing manual burden [29]. Such innovations demonstrate the potential of thoughtfully designed technology to not only support but amplify the benefits of structured disposition documentation.

Legal, regulatory, and accreditation implications

Structured discharge documentation also plays a crucial role in meeting legal, regulatory, and accreditation standards in hospital care. Miscommunications during hand-offs have been linked to medical errors and preventable adverse events; a landmark I‑PASS study demonstrated a 23 % reduction in medical errors and 30% reduction in preventable adverse events following implementation of a structured handoff bundle [30]. Moreover, a randomized controlled trial of a reengineered hospital discharge process showed a 5.8 % absolute reduction in 30-day readmission rates, consistent with Medicare expectations for safe discharge planning [16]. From a legal standpoint, inadequate documentation of communication about discharge planning with patients and families has been identified as a contributing factor in malpractice litigation; stronger discharge documentation may help mitigate liability risk [31]. Embedding structured discharge elements into daily notes can therefore help institutions comply with oversight standards while strengthening legal defensibility and operational integrity.

Patient-centered perspectives

In addition to operational and financial benefits, structured discharge documentation contributes to more patient-centered care by fostering shared decision-making and enhancing communication. Consistent, transparent communication of the discharge plan helps reduce patient anxiety, improve understanding of follow-up instructions, and strengthen trust in the care team, particularly among patients with limited health literacy [32]. Embedding daily updates about expected discharge timing, destination, and remaining barriers allows patients and families to actively participate in care planning and ask clarifying questions, which is associated with improved satisfaction and better adherence to post-discharge recommendations [33]. These updates can also be tailored for diverse populations by using multilingual templates and integrating interpreter services to ensure equitable communication for patients with limited English proficiency. These patient-centered benefits reinforce the value of structured documentation as a practical strategy to improve communication, empower patients and families, and support smoother transitions of care.

Case studies and practical examples

The impact of structured discharge documentation on patient outcomes has also been demonstrated in real-world hospital settings through targeted quality improvement initiatives. For example, a prospective study of a multidisciplinary discharge planning program in a large community hospital demonstrated significant reductions in 30‑day readmission rates and average length of stay after introducing structured daily discharge rounds and documentation templates [34]. Similarly, a large academic medical center implemented a standardized electronic discharge summary template, which led to measurable improvements in documentation completeness, timeliness, and physician satisfaction while reducing discharge delays [35]. These case studies underscore the practical feasibility of embedding structured documentation tools into daily hospital workflows and demonstrate that relatively simple interventions can yield measurable benefits in patient safety, care quality, and operational efficiency.

Economic and operational outcomes

Structured discharge documentation has also been associated with meaningful economic and operational benefits for healthcare systems. By reducing avoidable delays and facilitating timely discharges, hospitals can improve bed turnover rates and accommodate more admissions, thereby optimizing resource utilization and reducing opportunity costs [36]. Studies have shown that targeted discharge planning interventions significantly lower 30‑day readmission rates, which not only improves quality of care but also reduces costs associated with unplanned rehospitalizations, with some studies estimating savings of approximately $500-$1,000 per patient discharged [37]. In addition, standardized discharge processes have been shown to reduce hospital length of stay and prevent avoidable delays, producing measurable cost savings of up to $3,000 per patient episode for healthcare systems [38]. These findings highlight the role of structured documentation not only as a clinical quality measure but also as a strategic lever for operational and financial sustainability in modern healthcare environments.

Gaps in evidence and future research directions

Despite growing evidence supporting structured discharge documentation, important knowledge gaps remain that warrant further research. Many studies to date have been observational or single‑center, limiting the generalizability of findings to diverse healthcare settings [39]. Future research should include multicenter randomized controlled trials specifically designed to isolate the independent impact of structured dispo documentation on key outcomes such as length of stay, readmission rates, and patient satisfaction. Such trials could randomize hospital units or providers to structured documentation templates versus usual care, with predefined endpoints including time to discharge readiness, patient‑reported understanding of discharge plans, and clinician workload scores. Appropriate control strategies, such as cluster randomization and adjustment for baseline differences, would strengthen validity. In addition, more work is needed to understand the optimal integration of electronic tools, artificial intelligence, and decision support into discharge documentation workflows without increasing clinician burden [40]. Finally, research should examine the long‑term sustainability of such interventions and their cost‑effectiveness across different patient populations and care models. Addressing these gaps will help to refine best practices and ensure that structured documentation continues to evolve as an evidence‑based component of high‑quality, patient‑centered care.

## Conclusions

The “dispo in the progress note” represents a critical evolution in hospitalist documentation, transforming daily charting into a powerful tool for proactive discharge planning. By explicitly detailing key components such as the expected date of discharge, planned post-acute destination, consultant recommendations, patient and family communications, and identified barriers or delays, structured dispo documentation aligns the entire care team around clear and actionable goals. Evidence from both the United States and international experiences shows that embedding these elements in progress notes improves patient flow, reduces avoidable delays, enhances patient and family engagement, and mitigates regulatory and legal risks. Thoughtful implementation of dispo documentation also supports clinician workflow efficiency, minimizes redundant communication, and contributes to operational and financial sustainability. However, these outcomes may vary depending on hospital size, patient population, and the fidelity of implementation, and should be interpreted in light of these factors. As hospitals continue to prioritize safe, timely, and patient-centered discharges, incorporating these standardized components into daily progress notes should be regarded as a best practice that bridges communication gaps, advances quality improvement, and supports equitable care transitions. Future research should continue to refine these practices and explore innovative ways to optimize their impact on both patient outcomes and system performance.
